# Supplementary material for: A mixed method evaluation of a theory based intervention to reduce sedentary behaviour in contact centres- the stand up for health stepped wedge feasibility study
Source: PLoS One. 2023 Dec 15;18(12):e0293602. doi: 10.1371/journal.pone.0293602 (PMC10723690; doi:10.1371/journal.pone.0293602)
Supplement: S1 Protocol — (DOCX) [file pone.0293602.s002.docx]

#
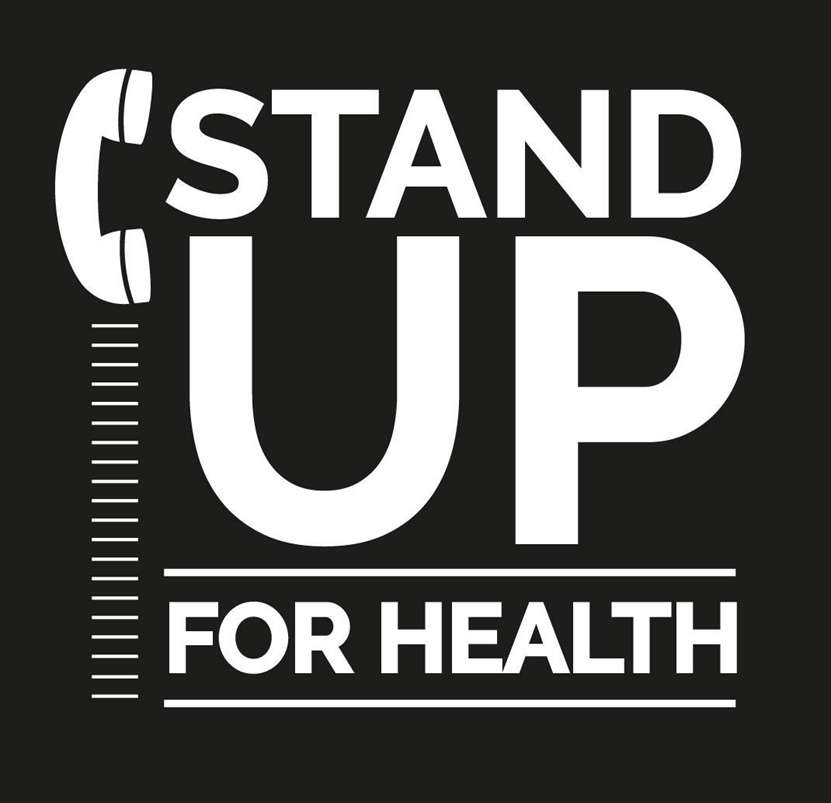


# STAND UP FOR HEALTH: A FEASIBILITY CLUSTER RANDOMISED CONTROLLED TRIAL (RCT) OF A THEORY BASED INTERVENTION TO REDUCE SEDENTARY BEHAVIOUR IN CONTACT CENTRES

# Background and Scientific Rationale

## Sedentary behaviour as a public health problem

Sedentary behaviour is a serious occupational health hazard, linked with an increased risk of type 2 diabetes, cardiovascular disease, musculoskeletal issues, and poor mental wellbeing [1-6]. These risks are independent of physical activity [7, 8]. Conceptually, sedentary behaviour and physical activity are different [9], with each thought to pose health risks independent of each other [5, 10-12]. A review of behaviour change strategies used for sedentary behaviour reduction among adults [13] reported that interventions which showed the most promise in reducing sitting time were those that aimed to changed sedentary behaviour rather than increase physical activity. The reduction of sedentary behaviour is therefore not a consequence of effectively promoting physical activity and should be recognised independently when developing interventions, guidelines and legislation.

Workplace sedentary behaviour is placing a large burden on employers and the healthcare system. Many employees working in office-based environments become exposed to prolonged periods of inactivity in static seated postures, which are enforced by factors such as ergonomic set-up and workplace culture [14]. This sedentary behaviour can impact significantly on the daily lives and activities of workers. For example, musculoskeletal issues are one of the most prevalent occupational health problems for desk based workers and are a leading cause for disability worldwide [15-17]. Estimates of the prevalence of musculoskeletal symptoms in computer users are as high as 50 per cent [18]. Lower back pain in particular is associated with prolonged sitting [19, 20].

## Factors contributing to sedentary behaviour in workplaces

In a traditional office setting, high levels of sedentary behaviour and low levels of physical activity are a result of multiple causal factors, which include workload and social norms surrounding workplace behaviours [21, 22]. Multi-level interventions have previously been successful in reducing total [23] and prolonged [24] sedentary behaviour within this setting, though few studies have been effective at promoting physical activity [25] in the workplace and only a limited number of trials have reported success in managing long-term positive behaviour change [24]. At present, only a very limited number of research studies have sought to explore the reduction of sedentary behaviour or promotion of physical activity in the contact centre setting.

**Contact centres and organisational drivers**

Many organisations representing the contact centre industry are highly constrained by profit-based drivers, cost minimization, and economic outcomes based on productivity and high quantity customer enquiry resolution [26]. This has resulted in standardised work methods, removing the need for specific skills and reducing costs associated with employee training. The work itself can be isolating, and employees may view themselves as replaceable. These factors can also often influence organisational investment into workplace health initiatives; fears of cost-ineffective programmes and reduced productivity rates are commonly presented by senior team leaders within private contact centres [27].

Whilst emergent technology and customer expectations are important for shaping the nature of the work contact centres (e.g voice recognition, more use of social media) there is no anticipation that this will remove the necessity for contact centre agents to use a computer of some sort. There may be a trend to more remote working in the next few years, but it is likely that sedentary will still be an issue.

## Contact centres as a setting for a public health interventions

There are over with 6,200 contact centres in the UK, employing 734,000 agents [28] which is roughly 1 in 25 of the UK workforce. Scotland and the North East of England are home to some of the largest contact centres in Europe. Workplaces are often considered as homogeneous, but there are wide variations in terms of the demographics of the worker, the amount of worker autonomy, salaries, the environment (e.g. rigid workstations) and culture.

The staff demographic profile is different from other non-manual workplaces, with a younger workforce, lower wages, and a higher proportion of women and part-time workers. The call handlers (the highest proportion of contact centre staff) earn an average salary of £16,319 per year compared with the national average of £26,500 which puts them in the bottom third of earners [29]. In terms of gender, they are more likely to be female (60:40) [28] and ONS [30] data shows a reasonably consistent picture of a 70:30 split of full and part time employment [31]. Career progression is limited and even the most experienced of contact centre staff earn only slightly more than those with least experience. Turnover rates are high in UK contact centres with average attrition rates of 21% reported in 2017. Short-term absence rates are also high with 6.8% of agent workdays being lost to short-term sickness and unauthorised absences [32].

**Contact centres and health**

Due to their occupational nature, contact centres are currently one of the most sedentary working environments, with some members of staff reporting up to 95% of their shift spent sitting. The technology in contact centres prevents staff from regularly leaving their desk and many call handlers often report stressful work environments due to low workplace autonomy, strict supervision of individual performance and commission-based salary systems [33]. One in four members of contact centre staff regularly experience musculoskeletal problems with 22.4% of sick days lost to such problems [30].  A recent study found that a common factor for sedentary behaviour shared by contact centre agents, team leaders and senior staff included a considerable lack of knowledge and awareness of sedentary behaviour as a risk factor for poor health in highly sedentary workplaces [27]. Additionally, there was also a low level knowledge among staff of guidelines and recommendations relating to sedentary behaviour and physical activity in the workplace and often there is no reflection of this in organisational policies [27, 34].

In 2012, a study found that working in a contact centre was associated with higher levels of sedentary behaviour than other office-based work [4]. Another study [35], hypothesised that the culture and policies of a contact centre may differ from other office-based work and thus present a unique setting.

As well as being a highly sedentary workplace, work in contact centres has been described as ‘constrained’ work, where opportunities for enrichment and intrinsic reward are apparently limited [26]. Contact centre agents have voiced concerns over job security, performance monitoring and a desire for increased autonomy over their working practices as influential factors for their motivation to participate in strategies to reduce sedentary in the workplace [27]. However, organisational pressures to maintain high levels of productivity and meet targets frequently work against organisational investment into health and physical activity programmes within some contact centres. This is often due to perceptions that these activities will reduce the agents’ call making time and lead to productivity losses [36]. One study reported that leaders and senior staff had ‘identified a conflict between promoting productivity and targets to call agents, while encouraging them to move more and sit less’ [27].

**Workplace intervention research to reduce sedentary behaviour**

Over the last 5-10 years there have been a number of systematic reviews of workplace interventions to increase physical activity and reduce sedentary behaviour [35, 37-39]. Most recently a systematic review of environmental interventions in workplaces (e.g. sit-to-stand desks) found evidence of significant reductions in sedentary behaviour in 14 out of 15 studies [38]. The most effective interventions were multi-component, targeting more than one level of the socio-ecological framework. In 2016, a systematic review assessed the effectiveness of white-collar workplace interventions to reduce sedentary time [39]. It similarly found that multi-component interventions had the greatest effect. Both reviews [38, 39] recommended a need to assess whether policy-based measures or organisational change could further increase effectiveness. One study assessed the effect of sit-stand desks and ergonomic awareness on reducing sedentary behaviour in 15 Swedish contact centres [40] and found that working at a sit-stand desk was associated with a slightly greater reduction in sitting time as opposed to sitting at a non sit-stand desk. Regular interruptions to sitting time during the work day have previously been found to significantly reduce discomfort in the lower back and fatigue levels in overweight and obese office workers, without affecting productivity [41]. Another study found that the use of standing desks led to significant reductions in upper back and neck pain in office workers [42]. A recent study exploring barriers to participation suggested that, ‘barriers occurred at multiple levels of influence, and support the use of ecological or multilevel models to help guide future programme design/delivery’ [43].

**Multi-component workplace interventions**

Given the specified need to address cultural and organisational factors affecting workplace behaviour, we developed and piloted Stand Up for Health (SUH, the intervention in this current proposal).  There are a limited number of other similar interventions but the evidence is still sparse and only one study, currently on-going, is UK based. This UK study is NIHR-PHR funded and is currently examining the effectiveness of the SMArT Work & Life intervention aimed at reducing sitting time of office workers [44].  Whilst this intervention is similar to our intervention in some aspects, Stand Up for Health specifically considers aspects of organisational change, targets contact centres, and takes a systems-based approach [45]. This takes into account the complexity of the context, resources and assets of specific contact centres.  The Stand Up Victoria study in Australia also had a multi-component intervention [46, 47]. However, height adjustable workstations were a main part of the intervention. Whilst we envisage some contact centres may take up this option, we recognise that not all contact centres have the resources, or desire, to implement them.  A third study in Perth, Australia, used a similar participatory approach to SUH but had no theoretical basis, and was only assessed at a 12 week timepoint [48].

**Contact centre research**

Many existing practices in contact centres contribute to prolonged sedentary behaviour. Introducing smaller environmental changes to some of these practices can considerably encourage reduced sitting times. Recommendations emphasise the importance of regularly breaking up sedentary time, with desk-based workers being encouraged to accrue 2-4 hours of standing and light activity daily during working hours [49]. However, workplace cultures can often contribute to perceived judgements of behaviour, where staff may feel uncomfortable going against ‘sitting-norms’ [50]. These perceptions can often deter employees from engaging in less sedentary practices during the working day. Stand Up for Health is designed to have organisational change as a key component of the intervention.

**Relevant policy and practice**

Policy regarding sedentary behaviour lags behind the evidence. A recent study reviewed current national and international occupational safety and health policy documents (e.g. guidelines, legislation, codes of practice) for their relevance to occupational sedentary behaviour [34]. The review found that many workplace and jurisdictions had legal frameworks which established a duty of care for occupational health but discovered that no occupational health and safety authority had a policy specifically targeting occupational sedentary behaviour. Although some existing policies have aspects relevant to sedentary behaviour in the workplace, the authors identified a need to address the emergent hazard of excessive occupational sedentary behaviour by developing specific policies for this issue. They also highlight a need to support workplace-based initiatives which aim to minimise sedentary behaviour and associated risks.

A number of awards exist across the UK which are designed to recognise and encourage efforts made by organisations to improve health and wellbeing in the workplace. These include the North East Better Health at Work Award (BHAWA) [51] in England and the Healthy Working Lives award [52] in Scotland. Although these awards have a number of categories for health promotion that can help mitigate some of the negative outcomes of excessive sitting time, reducing sedentary behaviour as a specific outcome is not acknowledged.

**Importance of this research**

Current UK workplace legislation means that many members of staff in contact centres receive remedial ergonomic support as a mitigation measure to reduce existing musculoskeletal issues, *only after* a chronic or musculoskeletal condition has been diagnosed [53]. However, current practices, compounded by workplace culture, inhibit initiatives that encourage contact centre staff to reduce sitting time [54]. Combined with the lack of policies from authoritative bodies that are specific to sedentary behaviour it is important that workplaces take proactive steps to develop their own organisational policies which include, and promote, opportunities for reducing occupational sitting time [34]. Given that contact centres are amongst the most sedentary workplaces [55], and employees report higher levels of stress and depression compared with other desk-based work [56], it is key that preventative approaches are implemented.

This work is currently needed to ensure healthier working policies are distributed equitably across all workplaces, not just those which have more worker autonomy and better working conditions. Creating healthier contact centres may be more difficult to do than for other workplace settings, which is why such an intervention is necessary. Also, building the capacity to develop and measure workplace-based interventions for health in contact centres is vital for developing a stronger business cases to encourage and enhance organisational uptake and buy-in.

# Study Aims and Research Questions

## Study Aims:

*Aim 1:* To test the acceptability and feasibility of implementing the Stand Up for Health intervention in contact centres

*Aim 2:* To assess the feasibility of using a cluster randomised controlled trial study design

*Aim 3:*To scope the feasibility of a future health economic evaluation of Stand Up for Health

*Aim 4:* Consider previous aims under the context of covid-19

### Research questions for Aim 1

1. What is the acceptability, feasibility, and utilisation of the various components of the intervention in a range of contact centres?
2. Does the programme theory and process of implementing the intervention work as intended?
3. Does the programme theory/intervention need adapting and in what ways?
4. Are there differences in delivery of the intervention, between different contact centres? If so what are the reasons for these?

### Research questions for Aim 2

1. Is the study design (cluster RCT) feasible for a confirmatory trial of an intervention to reduce sedentary behaviour in staff working in contact centres?
2. How many clusters and participants per cluster are required for a confirmatory trial?
3. What is the recruitment rate of participants in each cluster and how many are lost of follow-up (e.g. due to staff turnover)?
4. Are the range of study procedures (e.g. recruitment strategies and outcome measurement tools) feasible for a future confirmatory trial?
5. Are there differences in aspects of study procedures (e.g. uptake) between different contact centres? If so what are the reasons for these?
6. What are the preliminary estimates of the variability of primary (reduction of sedentary

behaviour in the workplace) and secondary outcomes within and between contact centres?

### Research questions for Aim 3

Is it feasible to provide estimates of the cost-efficiency of Stand Up for Health from a) an NHS and personal social services (PSS) perspective and b) and employers perspective.

# Design and methods

Feasibility study with a cluster RCT design (to address Aim 2) combined with a process and qualitative study (to address Aim 1), and an economic component (to address Aim 3).  As the intervention is implemented in a workplace, it is not possible to randomise at the individual level; therefore a cluster RCT is the only option.  We have explored the relative advantages and disadvantages of the stepped wedge and the cluster parallel group designs as the two most appropriate options. After much discussion we have decided that the design will be an **incomplete cross-forward cluster randomised trial** (see **Figure 1**). Our study design is unusual because it is not a standard stepped wedge design (it has lots of incomplete sections), but it is also not a standard wait-list control design because there are many cross-sectional comparisons which may not always be present in a wait list control design. Similar designs are increasingly being used in evaluation research and involve random and sequential crossover of clusters from control to intervention until all clusters are exposed [57]. It can be considered as an extension of the parallel cluster trial with a baseline period. Such a design makes it possible to achieve a phased introduction of the intervention. It combines pragmatism with a robust design, and the way the study is conducted has much in common with the parallel cluster trial. Such designs are considered to be appropriate when: 1) there is a belief that the intervention will be of benefit and unlikely to do any harm; 2) evaluating an intervention that will be implemented irrespective of evidence for effectiveness; or 3) when it will be logistically implausible to roll out the intervention simultaneously to all clusters [58]. This is an important consideration, since it would be very expensive to have enough equipment to loan out; we also wouldn’t be able to collect activpal data on half of the anticipated sample at one time-point. This design allows us to have a smaller number of individuals at each baseline assessment point thus smaller number of activpals and equipment and other resources required.

Whilst there continues to be debate over the design and its limitations (e.g. [59]), some of which are valid, evaluations of public health interventions often have to be pragmatic and take into account the stakeholders and context of the intervention involved (in this instance the contact centres). We argue that we need a pragmatic option for a number of reasons. First, it could potentially cause delays in the evaluation process if we waited until all contact centres were at the same stage of readiness for implementation. A structural/location/organisation change in one contact centre could delay the process. Second, it would be more costly and resource intensive to implement the intervention and collect baselines data in all the contact centres at a single time point. We have requested two researchers, but due to the spread of locations in Scotland and North East England, this would make implementation and data collection difficult and increase the potential for failure. Third, contact centres are already wanting to implement the intervention when they hear about it. It is unlikely to cause any harm. It has been implemented in one contact centre for over a year and only positive benefits have been reported. Journals such as The Lancet Global Health and Trials are publishing the results of such designs [e.g. 60, 61].

Setting

Ten contact centres with more than 100 employees in Scotland and/or the North East of England. These two areas have the highest rates of contact centres in the UK, employing up to 6% of the workforce in these regions [62]. We will consider other areas of England if there is interest and to ensure we have 10 contact centres taking part.

## Study population

As this is a cluster RCT, contact centres rather than the staff employed in the contact centres (including managers, supervisors and call handlers) will be recruited. All staff will have the opportunity to participate in the intervention. For the evaluation components, staff will have the option of taking part on an opt-in basis.

## Recruitment

Ten contact centres will be recruited and we aim to have them recruited by month three of the project. All staff in the contact centres who have been working in the contact centre will be invited (via email through their contact centre) to be involved in the evaluation of the intervention, with the aim of recruiting 27 individuals per contact centre per data collection period for the outcome data (270 in total), and 6-8 individuals per contact centre for the qualitative data collection (60-80 in total). Staff who are interested in participating will be sent an information sheet and consent form. It will be made clear that participation, or non-participation will not affect terms of their employment.

Retention

The average annual turnover (attrition) of contact centre staff is around 24% vs 15% for other industries.  This high rate of attrition has implications for the retention and follow up of participants. The high turnover is partly due to the number of students and people looking for short term work. Whilst this is a problem for the evaluation, it maybe more positive for the success of the intervention; it has the potential to impact on a range of people, enabling them to engage with health promoting activities that may encourage the development of lifelong habits.

One of the aims of this study is to determine the retention rates for a future study.  We have a number of strategies:

1. Staff will be incentivised (£5-10 gift voucher) to complete baseline and outcome data assessments
2. If staff leave the contact centre we will explore what methods it may be possible to use to follow them up (e.g., post, email, telephone) and evaluate the most effective
3. We will record data on length of time employed in the contact centres. This data will help us determine whether turnover is higher at the beginning of the employment period (which could impact on retention rates)

## Randomisation

Randomisation will occur in month three. The unit of randomisation is contact centres. A computer-generated block randomisation algorithm will be used to randomly allocate each contact centre to start the intervention at one of five time points, three months apart. Randomisation in this way allows us to introduce the intervention to each site in an unbiased way unrelated to time or the particular circumstances of each site. It also helps ensure there is approximate balance on average across all the intervention start times in terms of participant or contact centre characteristics.

Steps to minimise bias

### Allocation

Allocation to trial arms will be performed after recruitment, consent and baseline data collection by the statistician who will be blind to the contact centre identity.

Contamination

Whilst it is possible that staff may move between contact centres allocated to different trial arms, we anticipate that this will have little impact. We will, however, attempt to measure/evaluate the extent to which this occurs by our follow-up attempts of contact.

## Sampling and sample size

All employees at a site are likely to take part in some or all of the intervention activities. However, employees will have the option of taking part in the research evaluation component. When we discuss the sample size, we are referring to the number of people taking part in the research evaluation (not those only taking part in intervention activities). The sample size and target difference are the same as another similar study that proposed a sample size of 160 per arm to detect a reduction in workplace sedentary behaviour of 45 minutes per day [49]. Since we will have 6 control and post-intervention cross-sectional comparisons (see **Figure 1**), the target sample size is 160/6=~27 per contact centre per data collection period. There are 10 contact centres so we aim to recruit at least 270 employees in total taking part in the research evaluation. An aim of this feasibility study is to test sample size assumptions and produce a more accurate sample size calculation for a future study.

# The Intervention

## Theoretical basis

The intervention is in part based on two main theories: Social Cognitive Theory [63] and the Social Ecological Model (SEM) [64]. The intervention also aims to create a sense of ownership to increase the likelihood of longer-term sustainability [65]. While the SCT addresses many personal determinants and socio-environmental factors, the SEM takes the proposed multifaceted approach one step further to consider not only the individual and interpersonal levels, but to also consider the intervention at the organisational, environmental, and group level and takes into account the interactions between each of these [66]. By targeting multiple levels of the workplace, Stand Up for Health will aim to foster an atmosphere that will create a social norm within the office community to be able to sit and stand within the workplace. The SEM justifies and predicts that Stand Up for Health’s multifaceted approach will be effective, acceptable, feasible and sustainable. We also take a systems based approach, by recognising that the implementation and sustainability of the intervention is dependent on how adaptive the control centre system is to change.

The causal factors of sedentary behaviour in the workplace are complex and numerous, therefore no single activity (e.g. standing desks, goal setting) is likely to create significant change on its own. This complex, theory-based, public health intervention has been developed using the 6SQuID framework [65] and addresses theories of change at the individual; social/cultural; environmental; and organisational levels. The intervention addresses the complex causal factors of sedentary behaviour by exploring the theories of change through a range of evidence-based activities (see **Table 1**). Whilst activities will vary depending on local context (e.g. office and work space set-up) there must be at least one activity from each theory of change included in the intervention.

As an adaptive intervention, the fidelity of the intervention is to the theories of change rather than being prescriptive about activities that catalyse change. To ensure transferability, it takes into account the specific system (the contact centre, how it organises its work and how SUH will fit into the system) and context (e.g. layout of the centre, work-time flexibility, budget and resources available). Additionally, it includes all employees from the start of development, with the aim of creating a social norm to be able to stand more at work. By gaining insight from contact centre staff about their specific needs, this approach is more likely to lead to a sustainable and effective intervention [65].

## Development of the intervention

The programme theory was developed through a comprehensive literature review and qualitative work in a pilot contact centre. The intervention was developed using the six steps in quality intervention development (6SQuID) framework [65].

### 6SQuID Step 1: Defining and understanding the problem and its causes

Evidence on the causes of sedentary behaviour in the workplace, and existing sedentary behaviour interventions in contact centres and other workplace were identified through comprehensive literature reviews and surveys in the pilot contact centre.

### 6SQuID Step 2: Identifying which factors can be modified

Based on reviews of the evidence-base and qualitative data collection, all factors leading to sedentary behaviour in the contact centre were identified to ascertain which of these were potentially modifiable. Modifiable factors with the greatest scope for change were considered in order to identify which specific factors should be targeted through intervention activities.  These are described in the Fishbone Diagram  (**Figure 2**).

### 6SQuID Step 3: Deciding on the mechanism of change (theory of change)

These factors were used to develop the programme theory (**Figure 3**) and underlying theories of change below (**Table 1**).

### 6SQuiD Step 4: Clarifying how the mechanisms of change will be delivered (theory of action)

During this phase a workshop was held at the test contact centre to introduce staff to examples of intervention activities and equipment for the workplace. The workshop activities were developed based on feedback from staff in the focus groups. Staff were asked to add to the list with their own ideas, and also prioritise the ones which they wanted to try out.  The research team fed back the results to the SUH implementation group that then decided the final intervention activities to be implemented. There was at least one activity from each theory of change and they took account the resources, the assets (e.g. local spaces, existing equipment, spare spaces)  and budget available. Once the specific intervention activities were chosen the team worked with the contact centres to decide on an action plan for delivery and implementation of the activities (who, what, when, where).

### 6SQuID Step 5: Testing and adapting the intervention

Contact centre staff will be assisted and guided by the researchers during two wellness committee meeting to establish and set goals for the programme. Continuous feedback was gathered from staff throughout the duration of the intervention so that the delivery of activities can be adapted as necessary.

Results of the pilot

The pilot intervention is still being implemented and developed 18 months after it started. The contact centre has reported that 25% of staff now use standing desks (an increase from almost none). There are a number of different activities going on at any one time and the call centre staff are constantly thinking of new activities to implement. The contact centre also asked to ‘bolt on’ a mental health component - the research team worked with them to implement mindful activities which were either standing up interventions (e.g. jigsaw puzzles on a stand-up desk) or activities that encouraged them to leave their desk and be mindful (e.g. LEGO, knitting). This additional component is currently being implemented and has not yet been evaluated.

## The intervention to be delivered to contact centre staff

6SQUiD Stages 4 and 5 will form the basis of the tailored intervention for each contact centre. The hypothosised theories of change (Stage 3) remain constant but the theories of action are specific to each contact centre.  The initial stage of the intervention includes a ‘workshop’ that takes place at a contact centre, where staff have a chance to try out, suggest and vote for the activities they want to try out.  They also describe the environment, location, existing equipment and other assets which are then used to plan the activities. Some small pieces of equipment will be available to loan and try out through the SUH research team. Whilst the number of activities is not limited, there must be at least one activity from each theory of change. These activities are then converted into an action plan to be implemented over a number of weeks. A key intervention component is the creation of a SUH implementation group made up of all levels of contact centre staff who ensure that activities are implemented.  At the end of the intervention period a second workshop is held to check in as to which activities have worked and which haven’t. Further prioritisation and choosing of future activities is undertaken. All equipment and activities will be risk assessed, and details of how to use the equipment/undertake the activities provided. A website will be developed with useful resources, and opportunities for the contact centres to blog/share their experiences and create a community of SUH contact centres.

# Table 1.  Theories of change underpinning the intervention, and examples of activities

| **Theory of Change** | **Example of Activities*** |
| --- | --- |
| *Organisational* | SUH implementation group (made up of all levels of staff; this is the only obligatory activity); management led action plans; inclusion of non-sedentary behaviour as part of organisational strategies and goals; inclusion of non-sedentary behaviour activities for staff into roles and responsibilities (e.g. for supervisors); inclusion of ‘standing’ time into the working day. |
| *Environmental* | Equipment: Standing desks or a standing desk team area, bicycle desks, other equipment in communal places.  Repurposing or changing the environment: standing communal areas with mindful/enjoyable activities such as jigsaws or knitting, or a darts board; moving printers further away; exercise spaces; boards on walls to draw on. |
| *Social/cultural* | Group activities in work time such as five minutes of stretching per hour; group goal setting; exercise classes before or after shifts; competitions between workspaces or teams; workplace challenges; rewards for standing more/being more physically active; educational prompts. |
| *Individual behaviour* | Individual goal setting, active travel to and from work, lunchtime walks; apps; links to local groups and activities. |

*These are not exhaustive and the contact centre staff are encouraged to develop and think of their own activities.

### Duration of the intervention:

Duration of the intervention is defined as the period in which the centres take to develop their preferred activities for each theory of change, prepare an action plan for sustained engagement and test out some of the activities. This process is expected to take around three months, but may be dependent on the contact centre.

## Outcome measures

As this is a feasibility study we will be testing out the methods of collecting data on outcomes, as well as preliminary estimates of effectiveness. Table 2 outlines the methods/instruments used

# Table 2. Methods for Quantitative Data Collection

| **Outcomes** | **Measurement** |
| --- | --- |
| *Primary* |  |
| Sedentary time in workplace (objectively measured) | The activPAL™ is a small, thigh-worn device for assessing posture and is the preferred measurement instrument for assessing changes in occupational sitting [67, 68]. Participants wear the device continuously for seven days (during waking/sleeping hours and water-based activity) to provide adequate reliability [68, 69]. Following recommended procedures [69], we will isolate and determine changes in accumulated sedentary time whilst at work as our primary outcome. |
| *Secondary* |  |
| Sedentary time in workplace (subjectively measured) | The Occupational Sitting and Physical Activity Questionnaire (OSPAQ) will be used as a secondary measure given the favourable validity and reliability properties of this instrument [67, 68]. |
| Sedentary behaviour (overall) | The activPAL™ device to assess changes in:   - Prolonged sitting time in the workplace (bouts of ≥ 30 minutes) - Total sedentary time (i.e. including time outside the workplace such as at home and leisure time); workplace & total standing time; workplace & total sit-to-stand transitions |
| Physical activity | Physical activity will be assessed using both objective and subjective instruments.  Objective: activPAL™ device to assess changes over time in workplace and total stepping (utilising the stepping variable derived from the activPAL™ monitor)  Subjective: The International Physical Activity Questionnaire (long), last 7 days self-administered [68] will be used to quantity time spent in different domains of physical activity. This validated and reliable instrument assesses occupational, transportation, housework and recreation/leisure-time physical activity. |
| Productivity | Objective measures of productivity may include: absenteeism, presenteeism, call handling time, time spent talking, time spent on hold, time spent wrapping up a call, attendance, or sick leave. Subjective measures will be assessed using the Utrecht Work Engagement Scale and other measures in similar studies [69-70]. |
| Mental wellbeing | Warwick-Edinburgh Mental Well-being scale (WEMWBS) [71] |
| Musculoskeletal  health | Roland-Morris Disability Questionnaire: validated 24 item tool to measure back pain [72] |
| Activities | Questionnaires developed by the researchers to measure use and preference. |
| Staff turnover | Number of people leaving and number of new joiners over the study period (both in the contact centre as a whole and in the people taking part in the research) |

We will also collect data on demographics (age, gender, medical conditions which may impact on sedentary behaviour), activities use and preference; staff turnover.

### Data collection timepoints

In order to minimise cost and participant burden for this trial, and since the main purpose of the trial is to test study processes and procedures, data will only be collected at a maximum of three occasions per site: at the end of the control period, and at 3 and 6 months after the end of the intervention in each site. Sites 1 and 6 will not have a control period, and sites 5 and 10 will not have a post-intervention period, in order to minimise the duration of the study for which there is no concurrent vertical comparison between control and post-intervention periods. To increase the response rate, participants will be paid a small amount (£5 end of control period and £10 for 3 months data) for taking part in data collection.

### Assessment of unanticipated outcomes

We will provide each contact centre with a sheet to record harms such as injuries as a result of using the equipment. The contact centre staff will be made aware of this and be asked to record any harms they think are a result of the intervention. We will ask them to detail the type of activity where the harm occurred, the type of harm (e.g. injury) and the date and time. We will collate this data from all contact centre every three months. We will also use the focus groups as a way of eliciting any unintended consequences.  Adverse events will also be a standing item on all Study Management and Study Steering Committee meetings.

Control/comparator group

Waiting list control – all 10 sites will receive no intervention for between 3 and 12 months after study set-up is complete; then they will receive the intervention.

### Statistical analysis

The statistician will be blind to the allocation of the contact centres. The analysis and presentation of results will adhere to CONSORT guidance [73], and a statistical analysis plan. The analyses will be exploratory to investigate if it’s feasible to fit such a model in a future trial. We will not interpret the results as confirmatory and it is not the primary analysis of the trial.

Sedentary time in the workplace and all continuous secondary outcomes will be analysed using linear mixed effects regression analysis; including site and participant as random effects; and adjusting for calendar time since start of study, season (spring, summer, autumn, winter), and an indicator variable for whether the intervention has been implemented or not as fixed effects. Using observed data from all participants, 95% CI of the intervention effect sizes will provide a guide to indicate the likely effect sizes that we will observe in a future study. Sensitivity analyses will be incorporated to explore the effects of missing data and staff turnover to aid the design of a definitive trial. The assumptions underlying the sample size calculation performed by a similar study [49] will be tested and compared to the estimates we calculate using real data from the feasibility trial (e.g. standard deviation, within-site correlation, intra-cluster correlation coefficient) to inform the power calculation for a future trial. The heterogeneity of quantitative outcomes across sites (e.g. estimated via intra-cluster correlation coefficients) and variability in intervention delivery and processes across sites is of particular interest. Information gathered from this feasibility study will help determine the feasibility of conducting a future multi-site study with a larger number of contact centres.

## Process evaluation and qualitative study: (Aim 1)

We will use the **RE-AIM** framework (Reach, Effectiveness, Adoption, Implementation, Maintenance) as a framework to guide the process evaluation. RE-AIM was developed to translate research into practice [74]. We will use both quantitative and qualitative methods to explore the elements of RE-AIM:

**Reach:** the number of people (and %) in the contact centres who a) take part in one or more activities in the various components of the intervention; and b) take part in the research.

**Effectiveness:** this is not the primary outcome but we will be measuring whether there are indicators of effectiveness using the outcome measures described earlier

**Adoption:** we will explore through focus groups and talking with key stakeholders the degree to which the intervention was adopted. We will also measure engagement in the two workshops, and use of the website and other resources.

**Implementation:** we will be in regular contact with the SUH implementation group in the contact centres to assess which activities were implemented by the contact centres and why; whether all the theories of change had a corresponding activity which was implemented.

**Maintenance:** maintenance is a key feature of the intervention, so we will speak to contact centre staff (particularly the SUH implementation group) after 9 months (in contact centres that received the intervention early on) to assess the degree to which the activities have been maintained or dropped.

### Qualitative study

We will conduct an in-depth qualitative programme of research comprising of focus group discussions with a representative sample of the contact centre workforce and individual interviews with key stakeholders (i.e., members of the SUH Implementation group. Primarily, the qualitative component of our evaluation seeks to gather views and experiences of the SUH intervention activities, and implementation processes with a view to refining the Theories of Change (see Table 1) and the overall Programme Theory for SUH (see Logic Model upload). With contact centre workers, we will look to elucidate the specific mechanisms of change describing potential casual mechanisms for how SUH activities could impact on the specified short- and medium-term outcomes such as a reduction in sedentary behavior at work. We will also seek insight into why the SUH activities may not have worked as intended and any unintended consequences of the intervention. With key stakeholder interviews we will look to explore whether the process if implementation worked as intended across the different levels of the intervention activities.

The proposed study methodology will also be used for triangulation of quantitative evidence and stakeholder perceptions across multiple organisational structures, sectors and job roles. This component of our evaluation will contribute to enhancing the acceptability, reliability and replicability of future interventions in this setting. Given that there is considerable variation across the implementation of current workplace-based health policies and initiatives, this research will look to explore the generalisability of findings and identify any alternative factors influencing sedentary behaviour and physical activity in the contact centre setting. We will also explore whether and why there are differences in aspects of study procedures (e.g. uptake, retention) between different contact centres and the reasons for these. A key outcome of this study will be to develop and refine the programme theory which would be used to help guide the evaluation of any subsequent effectiveness trial.

### Sampling

Sampling will be purposive to ensure broad representation based on amount of involvement in the activities, as well as gender, age and staff member (e.g. manager, supervisor or call handler).

### Data collection

One focus group will be conducted at each intervention site (10 groups in total; approx. n = 60 individuals) with contact centre staff that took part in the activities and the research Additionally, one individual interview will take place in each site with key stakeholders involved in implementation. These will take place after 5 months. This will give contact centres time to implement the intervention fully, and participants time to try out the activities. The topic guide (focus groups with participants) and interview schedules (stakeholders) will be based around the programme theory exploring where appropriate; views and experiences of the intervention activities (and implementation of these) and impact on short- and medium-term outcomes. Additionally, we will collect qualitative data on key components of the RE-AIM framework; adoption, implementation and maintenance.

### Data analysis

Data will be transcribed verbatim, anonymised, and coding will be performed by at least two members of the research team. Codes will be developed based on RE-AIM and the programme theory. Analysis will be based on the elements of RE-AIM and the programme theory and will be coded for both inductive (emergent themes) and deductive (data driven themes). We will examine divergence and similarities across the contact centres to develop a comprehensive understanding of how the theories of change operate across a range of contact centres and also assess the mechanisms of change, implementation, sustainability and potential for a larger effectiveness study.

## Development of a health economic study (aim 3)

The health economic component of the study (Research question 8) will aim to lay the ground work for a health economic component of any future definitive trial, from a) an NHS and PSS perspective (Following the NICE reference case [75], and b) an employer’s perspective (which may be important at an implementation stage to leverage commercial support). With regards to a), while it is possible that fitness level improvement may be observable within a trial, it is not anticipated that such fitness would be likely to translate into changes in “hard” health outcomes or patterns of healthcare utilisation (ie primary or secondary care utilisation) within an observable trial period, hence standard within trial analysis is not appropriate. Instead the health economic component of the study will focus on scoping the possibility for future economic modelling of longer term outcomes through consultations with managers and clinicians regarding possible model structures and associated data sources, with targeted literature searches for potential parameter sources where identified. If appropriate this may be extended to include employer perspective outcomes. Within trial observations will be limited to estimates of the cost of direct implementation costs (such as workstation adjustments, information sessions or similar) presented alongside descriptive statistics regarding the utilisation of each, and measures of workplace productivity reported. The latter will be determined through consultation with local management to identify measures most useful to the participating sites, though it is anticipated that these will include most of those outlined in Table 3.

| **Outcome** | **Measure** |
| --- | --- |
| Absenteeism | WHO HPQ absenteeism and presenteeism questions |
| Presenteeism | WHO HPQ absenteeism and presenteeism questions |
| Productivity | Could include (depending on the contact centre): call handling time, time spent talking, time spent on hold, time spent wrapping up a call . |
| Quality of Life | EQ-5D |

## Progression criteria

We will proceed to a future larger study if all of the following are satisfied:

1. 95% confidence intervals for the primary outcome includes a clinically relevant reduction in sedentary time of 45 minutes per day or greater in favour of the intervention. This would reflect substantial progression towards accumulating recommended quantity of 2h/day standing/light activity during working hours [54] for employees in predominantly desk-based occupations.
2. Intervention successfully delivered in at least five of the sites within the study period, if at least one person in each site was able to use/experience at least one activity.
3. At least 10% of employees at a site were able to use/experience at least one of the intervention activities in the sites at which the intervention was successfully delivered.
4. Out of all the participants using/experiencing at least one of the intervention activities, primary and secondary outcome data was collected in at least 75% of participants overall.
5. Contamination between sites is low or else it is envisaged that contamination can be addressed in the study design of a future study.
6. It is envisaged that any practical difficulties in delivering the intervention across multiple sites or in measuring effectiveness can be overcome when conducting a future large-scale study.
