# Supplementary material for: A mixed method evaluation of a theory based intervention to reduce sedentary behaviour in contact centres- the stand up for health stepped wedge feasibility study
Source: PLoS One. 2023 Dec 15;18(12):e0293602. doi: 10.1371/journal.pone.0293602 (PMC10723690; doi:10.1371/journal.pone.0293602)
Supplement: S1 File — (DOCX) [file pone.0293602.s003.docx]

**S2- Stand Up for Health programme details**

Stand Up for Health (SUH) is an adaptive, multi-component intervention that aims to reduce sedentary behaviour in contact centres. Through a rigorous development process based on 6SQuID (1, 2), a theory of change was developed for the programme (3). The levels of change that need to be targeted to created sustained change are:

- Organisational
- Environmental
- Social
- Individual
- Ownership and contexts
- Information and awareness

The fidelity of the SUH intervention is to the theories of change. Rather than being prescriptive about activities that catalyse change, the SUH team would work with each contact centre to develop a customised theory of action. Examples of activities at each level of change can be seen in Table 1.

**Table 1. Examples of activities at each level of change**

| **Level of Change** | **Examples of activities** |
| --- | --- |
| Organisational change | •SUH committee •Action plan •Included in induction •Changes to working routine •Supervisor buy in |
| Environmental change | •Equipment from SUH team •Placement of equipment and designated SUH spaces •Changes to desk structure |
| Group activities | •Charity run/walks •Walking/running groups •Team-based activities •Competitions •Yoga/Tai Chi classes •Bingo |
| Ownership and context | •Initial event prioritising outcomes •SUH committee includes staff from all levels •Making sure all activities are fit for purpose |
| Information | •Website •Delivery of messages |
| Individual behaviour | •Motivational techniques- goal setting (weight loss, fitness, steps) •Desk-based stretches •Token system (where individuals get tokens for meeting targets and work towards centre goals) |

In each individual contact centre, the following process was adopted to develop and implement activities during the pre-lockdown period:

**Two workshops conducted to co-produce activities for each centre**

The SUH team conducted two workshops as part of the programme.

**Workshop 1:** The first workshop was delivered at the centre. Contact centre staff had the opportunity to try out various equipment and activities. Staff also participated in the prioritisation exercise, in which they used stickers to express their preferences for individual, social and environmental activities. The SUH team lent several pieces of equipment (e.g. exercise bike, stepper, twisting disks, mini table tennis and mini golf) to the centres, keeping in mind environmental factors and staff preferences. In addition, an office wellness company (Sit–Stand.Com®; Coalville, UK) provided desk risers to centres at no cost. The SUH team had a discussion with centre coordinators regarding the best style of desk riser for their contact centre, and the following were then shipped to the centres by Sit–Stand.Com:

Centre 2 – Yo-Yo Desk Slim 80 cm (× 2) and Yo-Yo Desk Mini (× 1)
Centre 3–Yo-YoDesk90cm(×2)andYo-YoDesk120cm(×2)
Centre 7 – Yo-Yo Desk Slim 80 cm (× 3)
Centre 9 – Yo-Yo Desk Slim 80 cm (× 2) and Yo-Yo Desk Mini (× 1)
Centre 10 – Yo-Yo Desk Slim 80 cm (× 2) and Yo-Yo Mat Medium (× 2)

Centre 11 – Yo-Yo Desk Slim 80 cm (× 2)

The SUH team worked with contact centre managers to understand the context (e.g. centre layout, work- time flexibility and shift patterns) and resource availability (budget, space, online material, equipment and staff members with physical activity or other expertise). The team used a resource assessment template to map out the assets and resources for the centre (Figure 1).

Figure 1. Resource assessment template


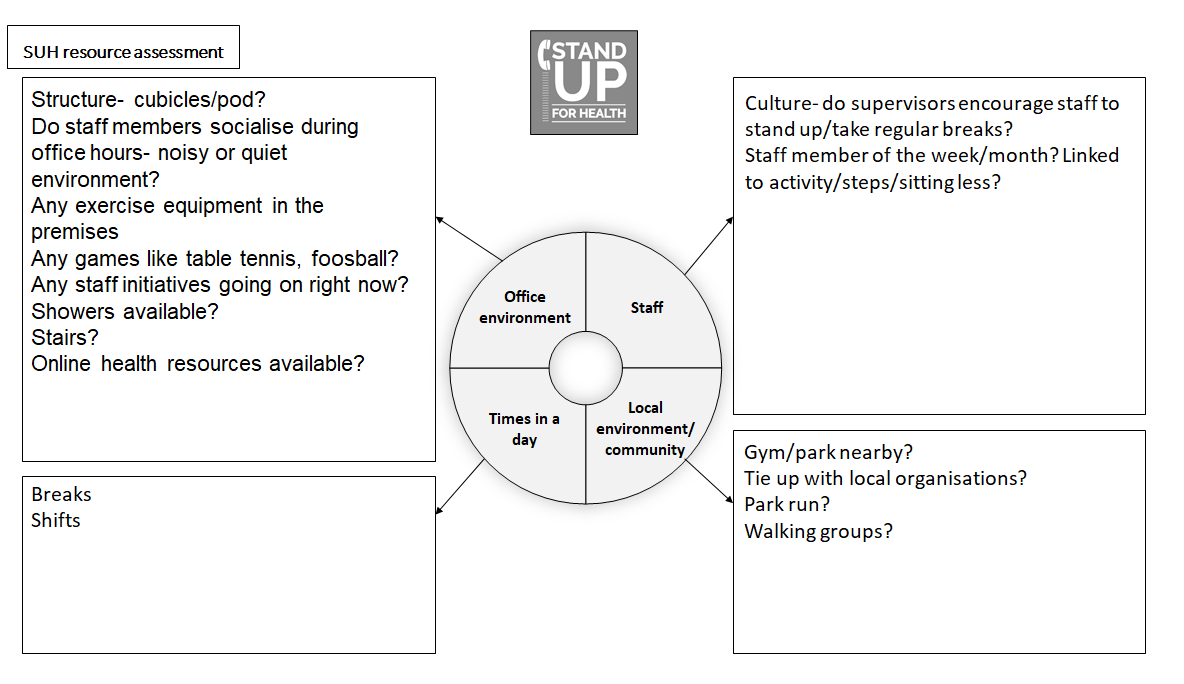


Reproduced from the SUH NIHR report (4)

**Workshop 2:** The SUH team returned to the centre for Workshop 2 after 3 months. During the second workshop, they spoke to the staff about activities that had been implemented, likes and dislikes, and suggestions to ensure staff involvement and ownership. During the second workshop, the SUH team would also retrieve any equipment lent to the centre. Having tried different equipment for 3 months, the centre would have an idea of what worked for them, and could make decisions on what they would like to buy for future use.

**Stand Up for Health committee**

Setting up a SUH committee was an important element of the SUH programme. It was recommended that a SUH committee be set up in each participating centre, consisting of staff members from different teams and roles. The SUH committee was responsible for procuring and generating ideas for activities from staff and aiding implementation of the intervention.

**Action plan**

After the initial workshop, the SUH team worked with the centre coordinator to develop an action plan specific to the centre. The SUH team encouraged the adoption of at least one activity from each level of the theory of change. A ‘SMART’ (specific, measurable, achievable, relevant and time-bound) approach was adopted to enhance success of implementation. The second workshop helped to refine the action plan.

**References**

1. Jepson R, McAteer J, Williams A, Doi L, Buelo A. Developing Public Health Interventions: A Step-by-Step Guide: SAGE Publications Ltd; 2022.

2. Wight D, Wimbush E, Jepson R, Doi L. Six steps in quality intervention development (6SQuID). Journal of epidemiology and community health. 2016;70(5):520-5.

3. Tirman L, Biggs H, Morrison K, Manner J, Sivaramakrishnan D, Baker G, et al. Stand Up for Health: Programme theory for an intervention to reduce sedentary behaviour in contact centres. Evaluation and Program Planning. 2021;89:102002.

4. Jepson R, Baker G, Sivaramakrishnan D, Manner J, Parker R, Lloyd S, et al. Feasibility of a theory-based intervention to reduce sedentary behaviour among contact centre staff: the SUH stepped-wedge cluster RCT. Public Health Research. 2022;10:13.
