# Supplementary material for: A mixed method evaluation of a theory based intervention to reduce sedentary behaviour in contact centres- the stand up for health stepped wedge feasibility study
Source: PLoS One. 2023 Dec 15;18(12):e0293602. doi: 10.1371/journal.pone.0293602 (PMC10723690; doi:10.1371/journal.pone.0293602)
Supplement: S2 File — (PDF) [file pone.0293602.s004.pdf]

Dear X,

Thanks again for attending the consult session, and providing valuable feedback on your work experiences and sharing your interests with us.

Here is some information and ideas based on what you told us.

Introduction

What is sedentary behaviour and how is it different from physical activity?

Sedentary behaviour is any behaviour where you are awake and sitting, reclined or lying down. Physical activity is any bodily movement produced by skeletal muscles that requires you to use energy.

What are the guidelines for physical activity and sedentary behaviour and why are they important?

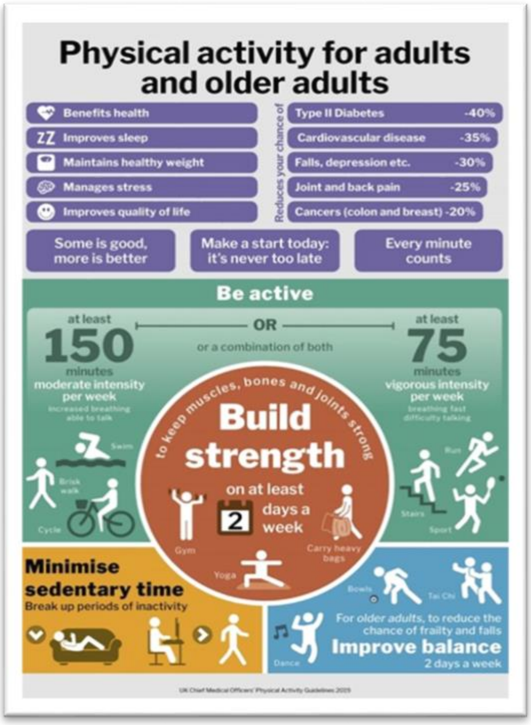

- At least 150 minutes of moderate intensity OR 75 minutes of vigorous intensity activity per week
- At least 2 days per week of strength-based activities/exercises
- At least 2 days per week of balance-based activities/exercises
- Minimise sedentary time by breaking up periods of inactivity (sedentary behaviour)

These guidelines are for both adults and older adults, and help to improve sleep, maintain healthy weight, manage stress, and improve overall quality of life. It can also reduce the risk of chronic conditions such as type II diabetes, cardiovascular disease, falls, depression, joint and back pain and some forms of cancer.

It is important to recognise the habits and behaviours that lead to increased sedentary time – you might not notice how much you sit during the day until you really think about it, especially if working from home.

How does Stand Up for Health (SUH) fit into this?

SUH aims to reduce sedentary behaviour and increase physical activity, and we hope this activity plan will help by giving you some ideas about how to go about this.

Recommendations

Based on your consult we found some resources, tools and/or activities that you might enjoy and could benefit you in helping to sit a bit less and move a bit more.

IMPORTANT NOTES:

- It's always a good idea to clear any exercises with your doctor before you begin a new regime, especially if you have a pre-existing health condition.
- Not all these resources are quality assured. Use at your own risk.
- The hyperlinks are not updated regularly, so if something no longer works, please inform Jillian on the SUH team at Jillian.Manner@ed.ac.uk. Don't worry - if links have changed, most of these resources can still be found quite easily through google.

Goal Setting

Use the attached template to set helpful goals for the week. A great start for you might be to use one of the suggested resources each day. For example, you could do one exercise video/app workout a day for an entire week and see how you feel. See 'exercise at home' below for ideas.

Mindfulness and Self Compassion

Michael Sealey	Guided meditations and hypnosis videos for various purposes.
The Honest Guys	Meditation channel with lots of guided meditations, and relaxation music.
Live the Life You love	Meditation channel with lots of guided meditations for various topics.
Positive Magazine Meditation	Meditation channel with lots of guided meditations for various topics.
Headspace	One of the leading meditation apps. 14 day trial. Student and family rates.
Calm	#1 app for Sleep, Meditation and Relaxation. 7 day trial.
Waking Up	Waking Up is a guide to understanding the mind, for the purpose of living a more balanced and fulfilling life.<br>Join Sam Harris—neuroscientist, philosopher, and New York Times best-selling author—as he explores the practice of meditation and examines the theory behind it.
Yoga for self-love with Adriene (video)	This 13-minute guided meditation is suitable for beginners and experienced meditators. Tune into the breath, honor your body, and lean into love
Meditation for anxiety with Adriene (video)	This 15 Minute Meditation For Anxiety guides you through a simple at home meditation to provide relief from anxiety, stress, and energetic imbalance.
Other Yoga resources	Yoga is a great way to calm the mind while strengthening and stretching the muscles. See Yoga resources here and on our website www.standupforhealth.co.uk

Use of the Stand Up for Health Website

Check out our website www.standupforhealth.co.uk to access a comprehensive list of online physical activity/sedentary behaviour resources and tips around exercise, healthy eating, snacking and more! As this is a private resource you will need to make an account to access it. Not to worry – signing up is easy, just click login in the top right corner of the homepage.

The forum can be also accessed on any smartphone through the Might Networks app. The app can be downloaded on the Google Play store or on the Apple App Store by searching for Mighty Networks.

Desk Exercises

Here is a short list of stretches/exercises which is organised by length of time and can be done on breaks. A more comprehensive list is available on our website.

Stretchwell Posture 4 Desk Stretch	Simple Exercises that can be done at your desk, whether it would be in the office or at home. (< 5minutes)
Stretchwell Lower Body 4	Simple lower body exercises that can be done at your desk, whether it would be in the office or at home. (< 5 minutes)
Standing yoga break	In this video, Dr Divya Sivaramakrishnan (from the SUH team) demonstrates some simple, low impact, standing yoga postures to break up your sedentary time. (5 minutes)
Yoga at your desk by Adriene	Yoga at your desk is perfect for anybody who spends much time working at a computer or in a cubicle (6 minutes)

We have also attached some desk stretches (pics with instructions) for you to try out.

Exercise at Home

We know are keen to make serious changes to your physical activity levels. Here is a short list of home exercise apps/websites we thought you might like. A more comprehensive list is available on our website. *Freeletics can also be used at the gym.

General	
POPSUGAR Fitness	Free access to over 500 workouts from celebrity trainers and fitness experts across strength, cardio, dance and yoga. They have made Active by POPSUGAR, a paid monthly subscription app with hundreds of workouts, free for a limited time!
The Body Coach TV (Joe Wicks)	The Body Coach TV is where Joe Wicks posts free weekly home workouts to help you get, stronger, healthier and happier. Sessions for children and older adults on here as well.
Fitness Blender	Website/app/Youtube channel with fitness videos, programmes and meal plans for all levels.
Les Mills On Demand	Les Mills classes offer a variety of at workouts for various fitness levels. 14 day free trial.

Fiit	Tonnes of recorded fitness classes of various lengths. ‘Netflix of fitness’.
**Yoga**	
Yoga with Adriene	Varying intensities of Yoga suitable for different time of the day and different difficulty levels.
Lululemon	Varied classes for different levels.
Down Dog: Yoga for Beginners	New to yoga? Yoga for Beginners is an entirely free app created to introduce absolute beginners to the practice of yoga. ***SUH team recommended***
**Programme Based**	
Freeletics	Freeletics is a sport where you train with your own bodyweight only. The training system combines High Intensity Training (HIT) and High Intensity Interval Training (HIIT) methods. A training session with Freeletics normally takes around 30 minutes and you have more than 1000 training variations to choose from. ***SUH team recommended***
Nike Training Club	Start training with a personal plan that guides you—while adjusting to your progress, schedule and other activities. With NTC, you get access to four 4–6 week training plans. Whether you have access to a full gym or are training from the comfort of your home, there's a routine that fits you and your schedule so that you can reach your goals.

We know that you are interested in various social activities, and also changes to the environment (such as changes to desk space and mindfulness activities) we suggested. We will feed that back to the centre.

Thanks again for taking part!

We would LOVE to hear from you so we can improve this service!

Please drop us a line to let us know if you liked something, have questions, concerns or would like to request something different.

Jillian.Manner@ed.c.uk or Divya.Sivaramakrishnan@ed.ac.uk
