# Supplementary material for: A mixed method evaluation of a theory based intervention to reduce sedentary behaviour in contact centres- the stand up for health stepped wedge feasibility study
Source: PLoS One. 2023 Dec 15;18(12):e0293602. doi: 10.1371/journal.pone.0293602 (PMC10723690; doi:10.1371/journal.pone.0293602)
Supplement: S3 File — (DOCX) [file pone.0293602.s005.docx]

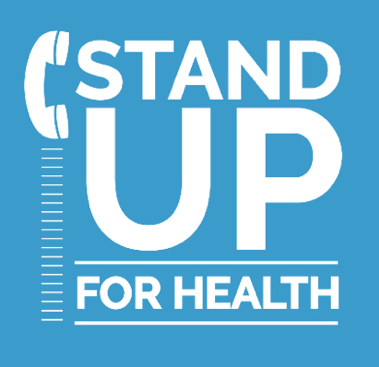


**Stand Up for Health**

**activPAL information and log booklet**

Name of participant:

Contact Centre:

activPAL Start Date:

Participant id (to be added by researcher):

Thank you for participating in the Stand up for Health study.

As a part of the study evaluation, you will be wearing an activity monitor. This booklet provides all the necessary information as well as contact information so that you can wear the monitor and provide us with data with minimal burden.

**Instructions**

**Wearing the activity monitor**

1. You have been fitted with an activity monitor (activPAL) and are requested to wear this for **7 days (24 hours a day)**.
2. **In case of skin irritation:** You should not experience any irritation, but in case you do, consider reattaching it to the other leg using the Tegaderm and alchohol pad provided. The instructions for reattachment are provided in page 2. If you feel uncomfortable with this, remove and cease wearing the activPAL.
3. **In case the activPAL becomes detached:** Please reattach the activPAL by following the instructions provided on page 2.
4. If you have any concerns, you can contact Jillian Manner or Divya Sivaramakrishnan at any time during working hours at 07748142970 or email them at info@standupforhealth.co.uk

**Filling in the log book**

1. At the end of each day , please record the following information in the log book for each of the 7 days that you will be wearing the activPAL:
2. The time you went to bed, and the time you actually fell asleep. Even if you don’t remember when you actually fell asleep, please give us your best guess.
3. The time you woke up, and the time you got out of bed.
4. The time you started and ended work.

1. On page 11 please tell us if the activPAL was detached and how.
2. On page 11, please record any additional comments you may have on wearing the activPAL, or relating to your sleep and work patterns.

**Instructions for reattaching the ativPAL**

1. With an alcohol prep pad thoroughly wipe down the area of the leg where the activPAL will be attached.
2. The activPAL is already covered in a waterproof sleeve and wrapped in one adhesive patch.
3. You will be attaching the activPAL to your right thigh.
4. Position the activPAL on the front of the right thigh, roughly one third of the way between hip and knee with the stick man standing up as shown in picture 1.
5. Peel the backing off the Tegaderm, and secure the activPAL to your thigh by placing the sticky dressing over the activPAL and your skin.
6. Peel off the top layer of the patch and smooth out the air bubbles and wrinkles as much as possible.
7. The activPAL is now attached.


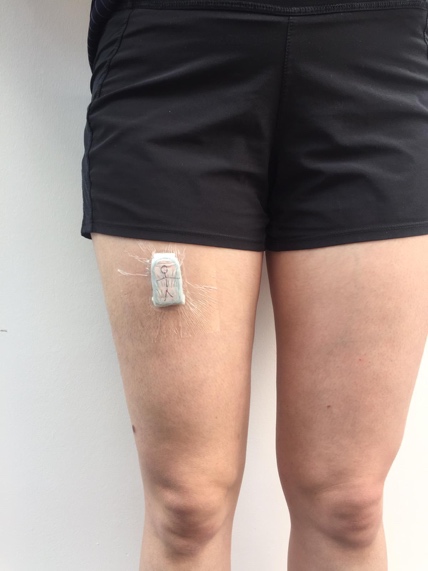


Picture 1. Position of activPAL

**Your Day 1:**

**Your Day 7:**

**Your activPAL removal date:**

**Instructions for return of activPAL and log books**

1. On day 7, we ask that you gently remove the activPAL from your thigh.
2. Gently peel off the Tegaderm, removing the activPAL from your thigh as it becomes free (note that this may cause some slight discomfort).
3. Continue to peel of the Tegaderm, until it is completely removed from your thigh.
4. Place the activPAL, the completed log books and any spare sheets of Tegaderm or alcohol pads back into the envelope. Close and seal the envelope.
5. Return the envelope to the SUH representative at your contact centre. It is important that you return the activPAL on time. The representative for your centre is:

**Please fill in the log books each day immediately before you get into bed to go to sleep.**

**Date log completed (Day 1): _______________**

dd/mm/yy

**Date when you last got out of bed: ________________**

Questions pertain to the time period since you last got into bed to go to sleep (for times please use the 24 hour clock):

What time did you get into bed? ___ : ___

What time do you think you fell asleep? ___ : ___

What time did you wake up? ___ : ___

What time did you get out of bed? ___ : ___

Did you go to work since you last got out of bed? (Please circle) Yes/No

If yes, please record the start and end of every shift-

Start time ___ : ___ End time ___ : ___

Start time ___ : ___ End time ___ : ___

Start time ___ : ___ End time ___ : ___

**Please fill in the log books each day immediately before you get into bed to go to sleep.**

**Date log completed (Day 2): _______________**

dd/mm/yy

**Date when you last got out of bed:________________**

Questions pertain to the time period since you last got into bed to go to sleep (for times please use the 24 hour clock):

What time did you get into bed? ___ : ___

What time do you think you fell asleep? ___ : ___

What time did you wake up? ___ : ___

What time did you get out of bed? ___ : ___

Did you go to work since you last got out of bed? (Please circle) Yes/No

If yes, please record the start and end of every shift-

Start time ___ : ___ End time ___ : ___

Start time ___ : ___ End time ___ : ___

Start time ___ : ___ End time ___ : ___

**Please fill in the log books each day immediately before you get into bed to go to sleep.**

**Date log completed (Day 3): _______________**

dd/mm/yy

**Date when you last got out of bed:________________**

Questions pertain to the time period since you last got into bed to go to sleep (for times please use the 24 hour clock):

What time did you get into bed? ___ : ___

What time do you think you fell asleep? ___ : ___

What time did you wake up? ___ : ___

What time did you get out of bed? ___ : ___

Did you go to work since you last got out of bed? (Please circle) Yes/No

If yes, please record the start and end of every shift-

Start time ___ : ___ End time ___ : ___

Start time ___ : ___ End time ___ : ___

Start time ___ : ___ End time ___ : ___

**Please fill in the log books each day immediately before you get into bed to go to sleep.**

**Date log completed (Day 4): _______________**

dd/mm/yy

**Date when you last got out of bed:________________**

Questions pertain to the time period since you last got into bed to go to sleep (for times please use the 24 hour clock):

What time did you get into bed? ___ : ___

What time do you think you fell asleep? ___ : ___

What time did you wake up? ___ : ___

What time did you get out of bed? ___ : ___

Did you go to work since you last got out of bed? (Please circle) Yes/No

If yes, please record the start and end of every shift-

Start time ___ : ___ End time ___ : ___

Start time ___ : ___ End time ___ : ___

Start time ___ : ___ End time ___ : ___

**Please fill in the log books each day immediately before you get into bed to go to sleep.**

**Date log completed (Day 5): _______________**

dd/mm/yy

**Date when you last got out of bed:________________**

Questions pertain to the time period since you last got into bed to go to sleep (for times please use the 24 hour clock):

What time did you get into bed? ___ : ___

What time do you think you fell asleep? ___ : ___

What time did you wake up? ___ : ___

What time did you get out of bed? ___ : ___

Did you go to work since you last got out of bed? (Please circle) Yes/No

If yes, please record the start and end of every shift-

Start time ___ : ___ End time ___ : ___

Start time ___ : ___ End time ___ : ___

Start time ___ : ___ End time ___ : ___

**Please fill in the log books each day immediately before you get into bed to go to sleep.**

**Date log completed (Day 6): _______________**

dd/mm/yy

**Date when you last got out of bed:________________**

Questions pertain to the time period since you last got into bed to go to sleep (for times please use the 24 hour clock):

What time did you get into bed? ___ : ___

What time do you think you fell asleep? ___ : ___

What time did you wake up? ___ : ___

What time did you get out of bed? ___ : ___

Did you go to work since you last got out of bed? (Please circle) Yes/No

If yes, please record the start and end of every shift-

Start time ___ : ___ End time ___ : ___

Start time ___ : ___ End time ___ : ___

Start time ___ : ___ End time ___ : ___

**Please fill in the log books each day immediately before you get into bed to go to sleep.**

**Date log completed (Day 7): _______________**

dd/mm/yy

**Date when you last got out of bed:________________**

Questions pertain to the time period since you last got into bed to go to sleep (for times please use the 24 hour clock):

What time did you get into bed? ___ : ___

What time do you think you fell asleep? ___ : ___

What time did you wake up? ___ : ___

What time did you get out of bed? ___ : ___

Did you go to work since you last got out of bed? (Please circle) Yes/No

If yes, please record the start and end of every shift-

Start time ___ : ___ End time ___ : ___

Start time ___ : ___ End time ___ : ___

Start time ___ : ___ End time ___ : ___

**Information on whether the activPAL was detached and reattached**

Did the activPAL become detached at any point over the 7 days of wear? Y/N

Date and time of detachment: Date __________ Time: __ : __

dd/mm/yy

Please provide a reason for detachment (please tick)-

- activPAL removed due to skin irritation
- Dressing peeled off
- activPAL removed due to discomfort
- Other- please specify ________________________________________________

Was the activPAL reattached? Y/N

Where was it reattached (please tick):  Right leg  Left leg

Date and time reattached: Date __________ Time: __ : __

dd/mm/yy

Please note down any additional comments or issues relating to wearing the activPAL for the last 7 days

Please note down any additional comments regarding sleep, work and physical activity patterns during the last 7 days

**Contact information**

If you have any concerns or questions, please contact Jillian Manner or Divya Sivaramakrishnan at 07748142970 or email them at info@standupforhealth.co.uk

In case of any complaints about the research: Please contact Professor Matthias Schwannauer, Head of School, School of Health in Social Science, [hos.health@ed.ac.uk](mailto:hos.health@ed.ac.uk), 0131 650 4327.

**Thank you for participating in this research project!**
