# Supplementary material for: A mixed method evaluation of a theory based intervention to reduce sedentary behaviour in contact centres- the stand up for health stepped wedge feasibility study
Source: PLoS One. 2023 Dec 15;18(12):e0293602. doi: 10.1371/journal.pone.0293602 (PMC10723690; doi:10.1371/journal.pone.0293602)
Supplement: S4 File — (DOCX) [file pone.0293602.s006.docx]

**S5- Progression criteria**

| **Progression criterion** | **Was the progression criterion achieved?** |
| --- | --- |
| 1. A 95% CI for the primary outcome includes a clinically relevant reduction in sedentary time of ≥ 45 minutes per day in favour of the intervention. This would reflect substantial progression towards accumulating the recommended quantity of 2 hours per day of standing/light activity during working hours for employees in predominantly desk-based occupations | No. For the pre-lockdown analysis, the 95% CI for the difference in daily sedentary time in the workplace was from –3.62 to 124.27 minutes greater for the intervention group. Therefore, given that this CI does not contain –45 minutes (indicating a reduction in favour of the intervention), this progression criterion has not been achieved. For the post-lockdown analysis, no device- measured sedentary time was measured. However, based on the OSPAQ (minutes sitting per working day), the CI was from –11.39 to 46.78 minutes, which, again, did not include the –45-minute reduction in sedentary time. Nevertheless, it should be acknowledged that data collection was impacted by Covid-19 and both the pre-lockdown and post- lockdown analyses were subject to potential bias |
| 2. The intervention was successfully delivered in at least five of the sites within the study period, and at least one person in each site was able to use/experience at least one activity | Yes. The intervention was successfully delivered in six sites pre lockdown and at least one person in each site was able to use/experience at least one activity. |
| 3. Primary and secondary outcome data were collected in at least 75% of participants overall. | Partially. Out of 155 participants recruited and assigned a participant ID pre lockdown, all of them provided at least some secondary outcome data and 152 (98%) provided valid data on the key secondary outcome of the OSPAQ. For the activPAL device-measured primary outcome, however, only 116 (74.8%) participants had primary outcome data collected and there were only 94 (61%) who recorded valid data suitable for analysis |
| 4. Contamination between sites is low or else it is envisaged that contamination can be addressed in the study design of a future study | Yes. None of the participants reported that they previously worked for a company that used the SUH intervention in either the pre-lockdown or the post-lockdown data collection periods. Although we accept that our pre-planned stepped-wedge design could not be fully achieved, based on the lack of evidence for contamination observed, we expect that contamination would be very low in a future cluster- randomised trial in this setting |
| 5. It is envisaged that any practical difficulties in delivering the intervention across multiple sites or in measuring effectiveness can be overcome when conducting a future large-scale study | Yes, provided that a future trial is designed differently. We think that the stepped-wedge design will be too difficult to achieve on a larger scale owing to the difficulties in maintaining site interest (e.g. if they are randomised to receive the intervention 12 months later) and ensuring that data collection takes place on schedule. Instead, we suggest that a parallel-group cluster-randomised trial in which sites are recruited in pairs (or groups) over time would be more practical. Detailed supporting information can be found in the full evaluation report (1). Findings related to acceptability of study design and data collection procedures will also be published separately. |

Adapted from SUH NIHR full report (1)

1. Jepson R, Baker G, Sivaramakrishnan D, Manner J, Parker R, Lloyd S, et al. Feasibility of a theory-based intervention to reduce sedentary behaviour among contact centre staff: the SUH stepped-wedge cluster RCT. Public Health Research. 2022;10:13.
