# Supplementary material for: A mixed method evaluation of a theory based intervention to reduce sedentary behaviour in contact centres- the stand up for health stepped wedge feasibility study
Source: PLoS One. 2023 Dec 15;18(12):e0293602. doi: 10.1371/journal.pone.0293602 (PMC10723690; doi:10.1371/journal.pone.0293602)
Supplement: S1 Fig — (DOCX) [file pone.0293602.s007.docx]

5 sites recruited out of a possible 11 contact centres

**Centre 6**

**Centre 10**

**Centre 11**

Baseline data collection (n=6)

Baseline data collection (n=7)

Baseline data collection (n=20)

Baseline data collection (n=18)

Baseline data collection (n=3)

3 months follow-up data but no baseline (n=2)

Baseline and 3-months follow-up data (n=4)

OSPAQ data available for pre-post analysis (n=4)

**Centre 7**

**Centre 3**

3 months follow-up data but no baseline (n=3)

Baseline and 3-months follow-up data (n=6)

OSPAQ data available for pre-post analysis (n=6)

3 months follow-up data but no baseline (n=4)

Baseline and 3-months follow-up data (n=7)

OSPAQ data available for pre-post analysis (n=5)

3 months follow-up data but no baseline (n=7)

Baseline and 3-months follow-up data (n=10)

OSPAQ data available for pre-post analysis (n=9)

3 months follow-up data but no baseline (n=1)

Baseline and 3-months follow-up data (n=1)

OSPAQ data available for pre-post analysis (n=1)
