# Supplementary material for: A mixed method evaluation of a theory based intervention to reduce sedentary behaviour in contact centres- the stand up for health stepped wedge feasibility study
Source: PLoS One. 2023 Dec 15;18(12):e0293602. doi: 10.1371/journal.pone.0293602 (PMC10723690; doi:10.1371/journal.pone.0293602)
Supplement: Attachment — Submitted filename: Response to reviewers.docx [file pone.0293602.s008.docx]

**Response to editors and reviewers**

**Journal requirements**

| **#** | **Comments** | **Author response** |
| --- | --- | --- |
| 1 | Please ensure that your manuscript meets PLOS ONE's style requirements, including those for file naming. The PLOS ONE style templates can be found at  <https://journals.plos.org/plosone/s/file?id=wjVg/PLOSOne_formatting_sample_main_body.pdf> and  <https://journals.plos.org/plosone/s/file?id=ba62/PLOSOne_formatting_sample_title_authors_affiliations.pdf> | We have formatted the manuscript in adherence with PLOS ONE's style requirements. We have made changes to the referencing format, formatting of headings, and naming of the supplementary files.  Unfortunately, we were unable upload separate files for the figures as these were not appearing in the pdf generated. We have hence included the figures within the main manuscript. We are happy to provide these files separately if requested. |
| 2 | We note that the grant information you provided in the ‘Funding Information’ and ‘Financial Disclosure’ sections do not match.  When you resubmit, please ensure that you provide the correct grant numbers for the awards you received for your study in the ‘Funding Information’ section. | We would like to edit the Financial Disclosure statement to ensure that it is the same as the Funding information. New Financial Disclosure:  This project is funded by the National Institute for Health Research (NIHR) [PHR project grant: 17/149/19]. The views expressed are those of the authors(s) and not necessarily those of the NIHR or the Department of Health and Social Care. https://www.nihr.ac.uk/  RP was partially supported in this study by NHS Lothian via the Edinburgh Clinical Trials Unit.  For the purpose of open access, the author has applied a Creative Commons Attribution (CC BY) licence to any Author Accepted Manuscript version arising from this submission.  The funders had no role in study design, data collection and analysis, decision to publish, or preparation of the manuscript. |
| 3 | We noted in your submission details that a portion of your manuscript may have been presented or published elsewhere. [A full evaluation report for the study has been published previously as required by NIHR- <https://doi.org/10.3310/IEXP0277>. We have acknowledged this in the paper and referenced any tables/figures adapted from the report. The manuscript has been developed and reviewed by the research team as a separate piece of writing suitable for publication in a journal, and includes several new figures, tables, and discussions. The presentation of results has also been modified to focus on specific research objectives. The paper has drawn out and presented a succinct summary of a rigorous outcome and process evaluation, and we believe that it would be relevant and useful to the research community.] Please clarify whether this publication was peer-reviewed and formally published. If this work was previously peer-reviewed and published, in the cover letter please provide the reason that this work does not constitute dual publication and should be included in the current manuscript. | A full evaluation report for the study has been published previously as required by NIHR- <https://doi.org/10.3310/IEXP0277>  There was a review process for the study report as per the NIHR process. However, the report is a 150 page document fulfilling the funding requirements. The current manuscript is a succinct summary of a rigorous outcome and process evaluation of a sedentary behaviour intervention in contact centres. The submitted manuscript contains several new figures, tables, references, and discussions. The authors are confident that after addressing the reviewer comments, the current paper would make a substantial unique contribution to literature. |

**Reviewer 1**

| **#** | **Reviewer comments** | **Author response and position in manuscript** |
| --- | --- | --- |
| 1 | The topic addressed is very interesting and relevant to the current reality. Undoubtedly, the nature of work often predisposes workers to sedentary behaviors, so it is an issue that should be addressed, given the consequences that this can have on people. | Thank you for your comment. |
| 2 | In the methods, with regard to the participants, a better characterization of these should be provided. | Thank you for prompting us to be clearer about participant characteristics. We had already provided demographic details of participants (Tables 4 and 5), but have now summarised these beforehand in the text as well (section – participant characteristics at baseline, page 23, lines 2-8, highlighted in yellow in the manuscript). |
| 3 | On the other hand, it is advisable to provide a solid justification, based on the available evidence, for the choice of the tools and/or instruments used. | Thank you for this comment. The study design and instruments have been discussed in the study protocol paper-  Parker et al (2020), <https://doi.org/10.1186/s40814-020-00683-1>  We aimed to be brief and avoid repetition of aspects that have been published in the protocol paper (Parker et al, 2020). |
| 4 | The article should provide more bibliographical support to the discussion. | We thank the reviewer for this comment. We have added 5 references to the discussion section. We hope the additional references have aided in strengthening this section. |

**Reviewer 2**

| **#** | **Reviewer comments** | **Author response and position in manuscript** |
| --- | --- | --- |
| 1 | It is necessary to deepen the methodology studied with respect to statistical issues (Bivariate, etc.), how to validate that within the results the study is acceptable and feasible, as well as to deepen more relevant conclusions, and lines of future work. | We have pre-specified our statistical methods in a published stats analysis plan (Parker et al (2020), <https://doi.org/10.1186/s40814-020-00683-1>). As it is a fairly long paper, we aimed to be as succinct as possible with methods, and did not want to repeat previously published material.  Although some of the methods needed to be modified because of difficulties implementing the design due to the covid-19 pandemic, we sought to stick as closely as possible to our pre-specified analysis plan. Analyses are mainly descriptive which is in keeping with the “feasibility” nature of the study design. The main purpose of the study was to assess feasibility of a future definitive cluster randomised trial and help inform the design of the future trial. This is also why we include a proposed sample size calculation for a future study. In the "Recommendations” section of the Discussion in the manuscript we have already provided detailed suggestions for “lines of future work”. |
| 2 | Introduction There are 10 citations for two lines, some sentences that are sentences end without reference example page 5 (Line n°1 and n°2) | For the first sentence in the introduction (page 3, lines 2-4), we have removed 5 citations, and retained the most relevant ones (highlighted in the paper).  For Page 5 (now lines 4-6), the sentence reflects our critical interpretation of the literature in the field and hence no references have been included. |
| 3 | I do not see the research questions and neither the hypotheses. | The research aims and objectives have already been reported in the Introduction section (page 6, line 18 till page 7, line 6).  As this is a feasibility study, we did not test any formal hypotheses. |
| 4 | It is important to see if there are studies in the country, and the occupational sector where the study was carried out and to name it. | Previous work on this topic, including studies conducted in the UK have already been summarised in the introduction (from page 4, line 4 to page 5, line 6). We have now emphasised the countries where the studies were conducted (highlighted in the text) and have also expanded this section to include the following studies:   - Chau JY, Sukala W, Fedel K, Do A, Engelen L, Kingham M. More standing and just as productive: effects of a sit–stand desk intervention on call center workers’ sitting, standing, and productivity at work in the Opt to Stand pilot study. 2016;3:68-74. - Morton S, Fitzsimons C, Jepson R, Saunders DH, Sivaramakrishnan D, Niven A. What works to reduce sedentary behavior in the office, and could these intervention components transfer to the home working environment?: A rapid review and transferability appraisal. Frontiers in Sports and Active Living. 2022;4. |
| 5 | Theoretical framework. Cases of bibliography of service companies are mentioned. There is a lack of case studies on what is being studied in the country. | This comment is not very clear. If the reviewer could provide more clarity, we would be happy to address this appropriately. The theoretical framework has been detailed in the programme development paper (Tirman et al (2021), <https://doi.org/10.1016/j.evalprogplan.2021.102002>), and we have now mentioned this explicitly in the paper:  “The SUH programme was developed through a rigorous process using the 6SQuID intervention development framework, as discussed in an earlier paper (22)” (Page 5, lines 9 – 10)  The RE-AIM framework has been described in detail in the methods, section titled “Process evaluation RE-AIM framework”, page 15    The point on lack of studies conducted in the country has been addressed as a part of the previous point (point #4). |
| 6 | Methodology Regarding the control group to see the differences, I also lack papers that support this type of methodology, in addition to a greater type of analysis with bivariate techniques and/or multifactorial analysis. | As stated above, we were following a pre-specified statistical analysis plan as closely as possible. It was not appropriate to use multivariate and multifactorial analysis due to the small sample size and focus on assessing feasibility. As stated in our study design paper [Parker et al (2020), <https://doi.org/10.1186/s40814-020-00683-1> ]: “Since this is a feasibility study, the main aim of the trial is to assess feasibility outcomes rather than conduct confirmatory statistical analysis.” This decision is also supported in the literature. See for example the CONSORT extension to pilot and feasibility trials: Eldridge SM, Chan CL, Campbell MJ, Bond CM, Hopewell S, Thabane L, Lancaster GA. CONSORT 2010 statement: extension to randomised pilot and feasibility trials. BMJ 2016 Oct 24;355. |
| 7 | Results. The whole section is missing based on the methodology explained, there is not much coherence, there is a lack of order. | We disagree that the Results section is incoherent. We use subheadings that correspond with the methods section to guide the reader through the Results, starting with descriptive statistics, moving onto graphs, sample size calculation for a future study, and staff turnover, before covering the process evaluation results from the research. |
| 8 | Bibliography. Only 20 authors in the last 5 years of the 49 authors, it is very outdated, much more is missing. | After updating the bibliography, 25 of 50 papers are from the last 5 years (50% of the reference list). Approximately 80% of the papers are published from 2015 onwards (40 of 50). Only 5 references were published before 2010- most of which are seminal papers. We believe that the bibliography now includes relevant papers in the field, and provides strong support to the rationale and discussions in the paper. |
